# Intein‐based modular chimeric antigen receptor platform for specific CD19/CD20 co‐targeting

**DOI:** 10.1002/1878-0261.70146

**Published:** 2025-10-13

**Authors:** Pablo Gonzalez‐Garcia, Noelia Moares, Wenjie Yi‐He, Rosa Luna‐Espejo, Ricardo Fernandez‐Cisnal, Javier Ocaña‐Cuesta, Juan P. Muñoz‐Miranda, Antonio Gabucio, Cecilia M. Fernandez‐Ponce, Francisco Garcia‐Cozar

**Affiliations:** ^1^ Departmento de Biomedicina, Biotecnología y Salud Publica, Facultad de Medicina Universidad de Cadiz Spain; ^2^ Instituto de Investigación e Innovación Biomédica de Cádiz (INiBICA) Spain

**Keywords:** acute lymphoblastic leukemia (ALL), chimeric antigen receptor (CAR), immunotherapy, intein, modular CAR, non‐Hodgkin's lymphoma (NHL)

## Abstract

Development of chimeric antigen receptor T‐cell therapy has revolutionized the treatment of B‐cell malignancies, although challenges such as antigen escape and tumor heterogeneity often decrease treatment success. Modular CARs targeting multiple antigens have been proposed as an interesting solution to address these challenges by reducing the likelihood of tumor cells evading treatment through the loss of a single antigen. In this study, we present a new modular CAR platform, termed CARtein, which takes advantage of intein interactions to jointly target CD19 and CD20 antigens. We demonstrate that the CARtein system, which features a universal CAR signaling backbone that covalently binds to specific scFv‐intein recognition partners, generates fully active CARs. Functionality was validated using Raji cells and K562 cells expressing CD19 and/or CD20, observing significant T cell activation through NFAT and NFκB promoter activity and CD69 upregulation. Overall, our study lays the foundation for the establishment of a new way to target multiple antigens through a universal and inert CAR backbone with highly specific activation.

## Graphical abstract

CARtein is a modular CAR platform that uses split inteins to splice antigen‐recognition modules onto a universal signaling backbone, enabling precise, scarless assembly without re‐engineering signaling domains. Deployed here against CD19 and CD20 in B‐cell malignancies, the design supports flexible multi‐antigen targeting to boost T‐cell activation and reduce relapse driven by antigen escape, a common failure mode in CAR‐T therapy.

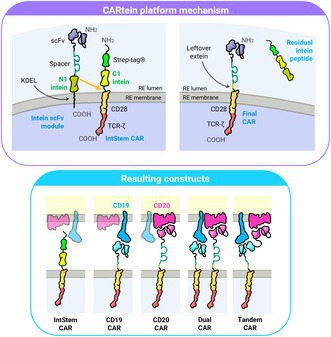

**Abbreviations**
ALL: acute lymphoblastic leukemia
CAR: chimeric antigen receptor
CAR‐T: chimeric antigen receptor T‐cell
CLL: chronic lymphatic leukemia
inteins: intervening proteins
IntStem: intein signaling CAR module
NHL: non‐Hodgkin's lymphoma
PEI: polyethylenimine
scFv: single‐chain variable fragments
scFvIRM: scFv‐intein recognition modules
TST: Twin‐Strep‐tag^®^
v/v: volume in volumen
VH: immunoglobulin variable heavy chain region
VL: immunoglobulin variable light chain region
VSVG: vesicular stomatitis virus glycoprotein

## 1 Introduction

Hematological cancers include multiple malignant neoplasms originating in the bone marrow and lymphatic system, thus encompassing a heterogeneous group of proliferative diseases, most of which have a dismal prognosis. During the last two decades, the breakthrough of chimeric antigen receptor T‐cell (CAR‐T) therapy led to a great advance in the treatment of these tumors, particularly B‐cell neoplasms, which account for one of the most frequent of all lymphomas and leukemias. In this regard, the CD19 antigen has been proposed as the main target for CAR‐T cells, thus being a major biomarker of lymphoproliferative diseases such as acute lymphocytic leukemia (ALL), non‐Hodgkin's lymphoma (NHL), and chronic lymphatic leukemia (CLL) [1, 2, 3, 4].

In these diseases, CAR‐T cell therapy has shown great success in the treatment of relapsed/refractory cases. However, a percentage of CAR‐T‐treated patients also relapse, which is often due to poor persistence of CAR‐T cells or to downregulation or loss of the CD19 antigen on the target cell membrane [5]. To overcome therapeutic failure due to antigenic escape, dual targeting has been proposed, whereby effector cells can still recognize leukemia/lymphoma B cells even if one of the antigens is lost. A proposed candidate antigen pair is CD19/CD22, as their simultaneous targeting has yielded great results in clinical trials, exhibiting a high remission rate after one month of treatment [6]. In addition, the dual CD19/CD20 target is recently gaining popularity as a candidate to prevent CD19‐loss‐induced relapse, as the CD20 target has been effective in CD19^−^ ALL cases [7, 8]. In this regard, CD20 expression levels vary depending on disease and patient‐specific conditions, and while higher levels correlate with better prognosis, lack of CD20 is associated with worse survival [9, 10].

Different strategies have been proposed to target multiple antigens. The most straightforward consists of administering two different CAR‐Ts, each targeting a different molecule, or else co‐transducing two different CARs in the same cells, although these mixed products are heterogenous and more expensive to manufacture. Alternatively, tandem CAR constructs harboring two different single‐chain variable fragments (scFv) linked together, each targeting its own cognate antigen, have also been assayed. Multiple CD19/CD20 tandem CAR‐T cells have been shown to exert cytotoxicity against malignant B cells, similar to CARs against individual antigens, although the functionality of these CARs depends on the relative position of each scFv with respect to the T cell membrane, which in turn depends on the distance of the antigen from the tumor cell surface [8, 11]. A very promising option is modular CARs consisting of an inert backbone to which soluble or *trans*‐expressed scFv can bind, allowing T cells to target multiple antigens through a single molecule. To date, this novel technology consists of various alternative adapter/receptor partners, such as biotin/avidin, leucine zippers, ULBP2/NKG2D, and SpyTag/SpyCatcher [12, 13].

In the present study, we propose to apply protein splicing to harness modular CARs. The proposed system takes advantage of inteins (*in*tervening pro*teins*), which are internal protein segments that self‐cleave from a larger precursor by a molecular reaction known as protein splicing, ultimately leading to the ligation of the flanking extein domains by a new peptide bond [14]. This reaction can occur as *cis*‐splicing, in a single molecule in which an intein is cleaved by an N‐ and C‐terminal extein (termed N‐ and C‐exteins, respectively), or as *trans*‐splicing, where an N‐extein/intein fragment reacts with a C‐extein/intein domain of another protein, both being independently translated. When these two parts are fused to the ends of protein modules, they form a split protein that undergoes a self‐splicing reaction whereby inteins are removed and protein modules are joined together, leaving behind a mature protein with a limited scar of only 6 AA [15]. These reactions have the additional advantage of being energy‐independent processes, thus requiring no assisting complexes or additional cofactors [16]. Therefore, a platform for modular CAR based on intein‐mediated binding of a recognition module to a universal CAR signaling backbone is an interesting alternative to the previously described modular CAR‐T cells, as this interaction occurs in a highly specific manner, binds both partners covalently, using a normal peptide link indistinguishable from the rest of the protein, and leaves minimal amino acids leftover. Thus, we developed a new system of modular CAR based on inteins to target multiple antigens while reducing the overall size of the construct. To this end, we engineered and transduced Jurkat cells with a second‐generation signaling CAR backbone (CD28/ζ) containing an inert extracellular intein‐Fc domain, which was co‐expressed with anti‐CD19 and/or anti‐CD20 scFv recognition domains, each bound to the complementary orthogonal inteins. In addition, a tandem scFv receptor against both antigens was also tested. This “CARtein” system, based on intein splicing, is a good candidate for CAR optimization and multi‐antigen targeting.

## 2 Materials and methods

### 2.1 Construction of CARtein, CD19, and CD20 expression plasmids

The versatile intein signaling module (IntStem CAR) to which different scFvIRM can be bound was codon‐optimized and synthesized by GeneArt™ (Thermo Fisher Scientific Inc., Carlsbad, CA, USA) with sequences encoding from 5′ to 3′ a hIgKVIII leader sequence, the Twin‐Strep‐tag^®^ (TST) peptide, a flexible interchain linker (GGGS)_3_, a IMPDH‐1 C‐intein‐extein domain (22) in frame with a CD28 transmembrane and cytoplasmic domain followed by a TCR‐ζ domains. The IntStem CAR coding sequence was cloned into the lentiviral shuttle vector pHR'SINcPPT CEW via a LR Gateway™ reaction, thus allowing its expression under a SFFV promoter [17].

Three different scFvIRM constructs (GeneArt™) were synthesized to serve as binding partners for the IntStem CAR module, based on the anti‐CD19, anti‐CD‐20 and tandem anti‐CD19‐CD20 scFvIRMs described by Zah *et al*. [18] with a Igκ leader. CDS for anti‐CD19 scFv (VL‐VH) was followed in frame by sequences coding for an IgG_4_ hinge domain, N‐intein IMPDH.1‐N, a flexible (GGGS)_2_ linker c‐myc and 6His tags and a (G_2_S)_2_A_3_‐KDEL sequence that allows scFvIRM to be sequestered in the ER until transplicing with the IntStem module occurs. The anti‐CD20 scFvIRM construct was engineered like anti CD19 but instead of the IgG_4_ hinge domain a longer spacer including hinge‐CH_2_‐CH_3_ was used, as anti‐CD20 CARs require a longer extracellular domain due to the position of CD20 target close to the tumor membrane [18]. In addition, a CD20(VL‐VH)‐(GGGS)_4_‐CD19(VH‐VL) tandem scFvIRM was also engineered with just the hinge spacer as CD19 itself correctly separates anti‐CD20 from the membrane to reach the shorter CD20 antigen [18].

For expression of the human CD20 antigen, a lentiviral expression plasmid encoding for human CD20 (pJRH‐1328 LV EF1a‐CD20 IRES‐EGFP; Addgene plasmid no. 201918), which also encodes for a bleomycin resistance gene, was directly packaged into lentiviral vectors. In the case of CD19, to express both CD19 and CD20 under resistance to different drugs, we generated an expression plasmid by cloning the CD19 coding sequence from a previously established plasmid (pJRH‐1363 LV EF1a‐CD19 IRES‐EGFP; Addgene plasmid no. 201919) into pLEX_307, [a gift from David Root (Addgene plasmid # 41392; http://n2t.net/addgene:41392; RRID:Addgene_41 392)] which allows for a strong CD19 expression under puromycin resistance. Both Addgene #201918 (http://n2t.net/addgene:201918; RRID:Addgene_201 918) and #201919 (http://n2t.net/addgene:201919; RRID:Addgene_201 919) plasmids were a gift from Jennifer Doudna [19].

### 2.2 Cell lines and cultures

All human cell lines were from American Type Culture Collection (ATCC, Manassas, VA, USA). To study T cell activation, a cell subline derived from Jurkat T JE6.1 (Jurkat, RRID:CVCL_0367) was used. This cell subline, known as Jurkat‐TPR (Prof. Steinberger, Medical University of Vienna), expresses the fluorescent proteins CFP, eGFP, and mCherry respectively governed by NFκB, NFAT and AP‐1 promoters, and has previously been used to assess CAR‐T cell activity [17, 20]. The human B‐lymphoma cell line Raji (RRID:CVCL_0511) were used as CD19^+^ CD20^+^ target cells. In addition, K562 cells (RRID: CVCL_0004), a chronic myelogenous leukemia cell line, were employed as parental cells to generate different single‐antigen sublines. Lastly, human embryonic kidney cells (Lenti‐X 293T, RRID: CVCL_4401) were used for lentiviral production. All cells were maintained at 37 °C and 10% CO_2_ in standard DMEM GlutaMAX media supplemented with 10% (v/v) FBS (Gibco), 10 mm HEPES, 1% (v/v) sodium pyruvate, 100 units·mL^−1^ penicillin–streptomycin, 2 mm l‐glutamine and 50 μm 2‐mercaptoethanol (Invitrogen, Carlsbad, CA, USA), except for K562 cells, which were maintained in Iscove's modified Dulbecco's medium (IMDM), with the same supplementation as for DMEM. All experiments were performed with mycoplasma‐free cell lines that had been authenticated in the last year by Secugen Inc. using short tandem repeat (STR) profiling with the AmpFLSTR Identifiler PCR Amplification Kit (Thermo Fisher Scientific), which interrogates CSF1PO, D2S1338, D3S1358, D5S818, D7S820, D8S1179, D13S317, D16S539, D18S51, D19S433, D21S11, FGA, THO1, TPOX, vWA, and the sex marker Amelogenin. Briefly, STR loci were amplified by PCR using fluorescently labeled primers, and labeled fragments were separated by capillary electrophoresis on an ABI 3730 instrument (Applied Biosystems). Allele sizes were analyzed, and genotypes assigned for each locus using GeneMapper software (Applied Biosystems). The resulting STR profiles were compared against reference entries in the Cellosaurus database (ExPASy) to verify cell identity and exclude cross‐contamination or misidentification.

### 2.3 Primary T cells

Peripheral blood samples were obtained from healthy donors following informed consent and in agreement with the Helsinki Declaration from June to July 2025 in the Puerta del Mar University Hospital (HUPR). Peripheral blood mononuclear cells (PBMCs) were isolated by density gradient centrifugation using Corning™ lymphocyte separation media, as per the manufacturer's instructions. Briefly, diluted blood was layered over the separation media and centrifuged to yield a buffy coat layer containing PBMCs, which was collected and washed with phosphate‐buffered saline (PBS). CD3^+^ T cells were then purified from the PBMC fraction by magnetic‐activated cell sorting (MACS) using CD3‐specific microbeads. The cell suspension was incubated with the beads, and the CD3^+^ fraction was isolated using a magnetic separation column according to the supplier's protocol. Purified CD3^+^ T cells were subsequently activated with the T Cell Activation/Expansion Kit, human (8·× 10^8^ PBMC or 4 T; Miltenyi Biotec), in the presence of interleukin‐2 (IL‐2, final concentration of 50–100 IU·mL^−1^) in complete culture medium. Cells were cultured at 37 °C in a humidified CO_2_ incubator and harvested for downstream applications. All experimental procedures were reviewed via submission through the Andalusian Biomedical Research Ethics Portal (PEIBA) and approved by the Coordinating Committee on Biomedical Research Ethics of Andalusia (Comité Coordinador de Ética en Investigación Biomédica de Andalucía, CCEIBA), license number SICEIA‐2024‐002038.

### 2.4 Lentiviral vector generation

Lentiviral supernatants were generated by transient co‐transfection of HEK Lenti‐XTM 293T cells with the different vector plasmids, pCMV∆R8.91 (RRID:Addgene_202687) and pMD2.G (VSVG) (RRID:Addgene_12259), as previously described [17]. Cells were added to collagen‐coated 100 mm plates in supplemented DMEM, 24 h prior to transfection. The following day, cells were transfected in OptiMEM™ medium (Thermo Fisher Scientific Inc.) with 20 μg of vector plasmid, 13 μg of pCMV∆R8.91, and 2.3 μg of pMD2.G by polyethylenimine (PEI)‐mediated transfection [21]. Supernatants were collected 48 and 72 h after transfection, and cells were removed by centrifugation. Lentiviruses were concentrated using the Lenti‐X™ concentrator (Clontech), and lentiviral pellets were frozen at −80 °C until use.

### 2.5 Cell transduction

A parental IntStem CAR‐TPR cell line was generated by transducing 3 × 10^5^ TPR cells in a 48‐well plate with the corresponding lentiviral pellet, and 40 μg·mL^−1^ Blasticidin S HCl (Thermo Fisher Scientific Inc.) was added 48 h post‐transduction. IntStem CAR expression was assessed by staining with Strep‐Tactin^®^XT DY‐649 (Iba‐Lifesciences, #2–1568‐050). Once this cell subline was stablished, 3 × 10^5^ IntStem CAR‐TPR cell aliquots were left untransduced or transduced with either tandem anti‐CD20‐anti‐CD19‐scFvIRM (Tandem CAR), anti‐CD19‐scFvIRM (CD19 CAR), anti‐CD20‐scFvIRM (CD20 CAR), or both anti‐CD19‐scFvIRM and anti‐CD20‐scFvIRM (Dual CAR). Puromycin (Thermo Fisher Scientific Inc.) was added at a concentration of 0.25 μg·mL^−1^ 48 h after transduction. The efficacy of the intein reaction between IntStem CAR and different scFvIRM partners was assessed by detecting the loss of TST expression with Strep‐Tactin^®^XT DY‐649, as well as the presence of the IgG spacer domain by staining with biotinylated goat anti‐human IgG antibody heavy chain (Antibodies‐Online Cat# ABIN375955, RRID:AB_10765281, Aachen, Germany). The presence of scFv was also assessed by staining with biotinylated Recombinant Protein L (ACROBiosystems). Both Protein L and anti‐human IgG were subsequently stained with PE‐conjugated streptavidin™ (Thermo Fisher Scientific Inc.).

Primary human T cells were transduced 48 h after activation in retronectin‐coated plates. Lentiviral preparations were thawed and resuspended in 500 μL of medium for each well, including those requiring co‐transduction with two viruses. For control wells, 500 μL of medium was added. Virus‐containing plates were centrifuged at 2000 ***g*** for 1 h at 32 °C to enhance viral adsorption. After centrifugation, 1.7 × 10^6^ activated T cells were added to each well in a final volume of 2 mL of complete culture medium supplemented with the appropriate amount of IL‐2. The virus and cell mixture was then centrifuged at 500 ***g*** for 5 min at 32 °C to promote transduction. Following this step, cells were collected and maintained in culture for further processing.

Two weeks after transduction, T cells were expanded by co‐culture with CD19 and CD20 expressing irradiated Raji cells at a 5:1 Effector:Target ratio. Irradtion 30 Gy was performed using a TrueBEAM linear electron accelerator [Varian Medical Systems, Palo Alto, CA, USA] in accordance with the manufacturer's guidelines and standard institutional protocols to ensure consistent and accurate dose delivery. Cells expressing only the signaling module and untransduced controls were stimulated with anti‐CD3 and anti‐CD28 activation beads. After three days of expansion, all cells were harvested and stained with protein L to assess mature CAR surface expression by flow cytometry.

K562 cells were transduced with lentiviral particles encoding for CD19 or CD20 to obtain individual cell sublines expressing individual antigens. 48 h after transduction, 0.25 μg·mL^−1^ Puromycin was added to cells transduced with CD19, and 100 μg·mL^−1^ Bleomycin (Thermo Fisher Scientific Inc.). to those with CD20. In addition, CD19^+^ CD20^+^ K562 (2Ag‐K562) cells were obtained by co‐transduction of both antigens and subsequent selection with both Puromycin and Bleomycin. CD19 and CD20 expression in all K562 sublines and in Raji cells was validated by labeling with human anti CD19‐PE (Cat# 6603024, RRID:AB_2716572 Beckman Coulter, Inc., CA, USA) and CD20‐APC (Cat# 375505, RRID:AB_2888856 Biolegend, San Diego, CA, USA) monoclonal antibodies previously treated with human FcR blocking reagent (Cat# 130‐059‐901, RRID:AB_2892112, Miltenyi Biotec) for 15 min at room temperature. Data acquisition was performed on a Cytek^®^ Aurora 5 L 16UV‐16 V‐14B‐10YG‐8R spectral cytometer (RRID:SCR_019826, Cytek, CA, USA).

### 2.6 T cell activation assay by spectral flow cytometry

Effector CAR‐T cells were cocultured with Raji cells in 96‐well plates at target:effector (T:E) ratios of 1:1, 1:5 and 1:10 in 5% CO_2_ and 37 °C for 24 h and 48 h. In addition, to test the specificity of each scFv toward its cognate target, effector cells were co‐cultured with WT, CD19^+^, CD20^+^ or 2Ag K562 cells at target:effector (T:E) ratios of 1:1 in 5% CO_2_ and 37 °C for 24 h and 48 h. In each case, cells were first counted and centrifuged to replace the consumed medium with fresh supplemented DMEM.

For each measurement, cells were washed twice in ice‐cold PBS and treated with FcR blocking reagent for 15 min at room temperature according to the manufacturer's procedure. Cells were then washed and labeled with anti‐human CD69‐APC (Cat# 310909, RRID:AB_314844) and CD3‐PerCP/Cy5.5 (Cat# 100218, RRID:AB_1595492) antibodies (both from Biolegend). To exclude dead cells and debris from analysis, they were stained with Zombie NIR™ Fixable Viability Kit (Biolegend) for 15 min at room temperature. Data were acquired on a Cytek^®^ Aurora 5 L 16UV‐16V‐14B‐10YG‐8R spectral cytometer, and the analyses were performed in FlowJo software V10.1 (RRID:SCR_008520, TreeStar Inc., Olten, Switzerland).

### 2.7 Statistical analysis

Data was previously evaluated with flowjo v10.8.1 software (TreeStar Inc., Olten, Switzerland). Then, statistical analyses and corresponding graphs were generated using GraphPad Prism v10.0 (RRID:SCR_002798, GraphPad, La Jolla, CA, USA). For comparisons between more than two groups, a two‐way ANOVA was used, and Bonferroni's *post hoc* test was performed to ensure the reliability of multiple comparisons. To compare differences between two groups, a two‐tailed unpaired Student's *t*‐test was performed. Values are expressed as the mean of triplicates ± SD, with significance levels marked in the figures (*P < 0.05; **P < 0.01; ***P < 0.001).

## 3 Results

### 3.1 Development of the CD19‐CD20 bispecific CARtein system

**Fig. 1:**
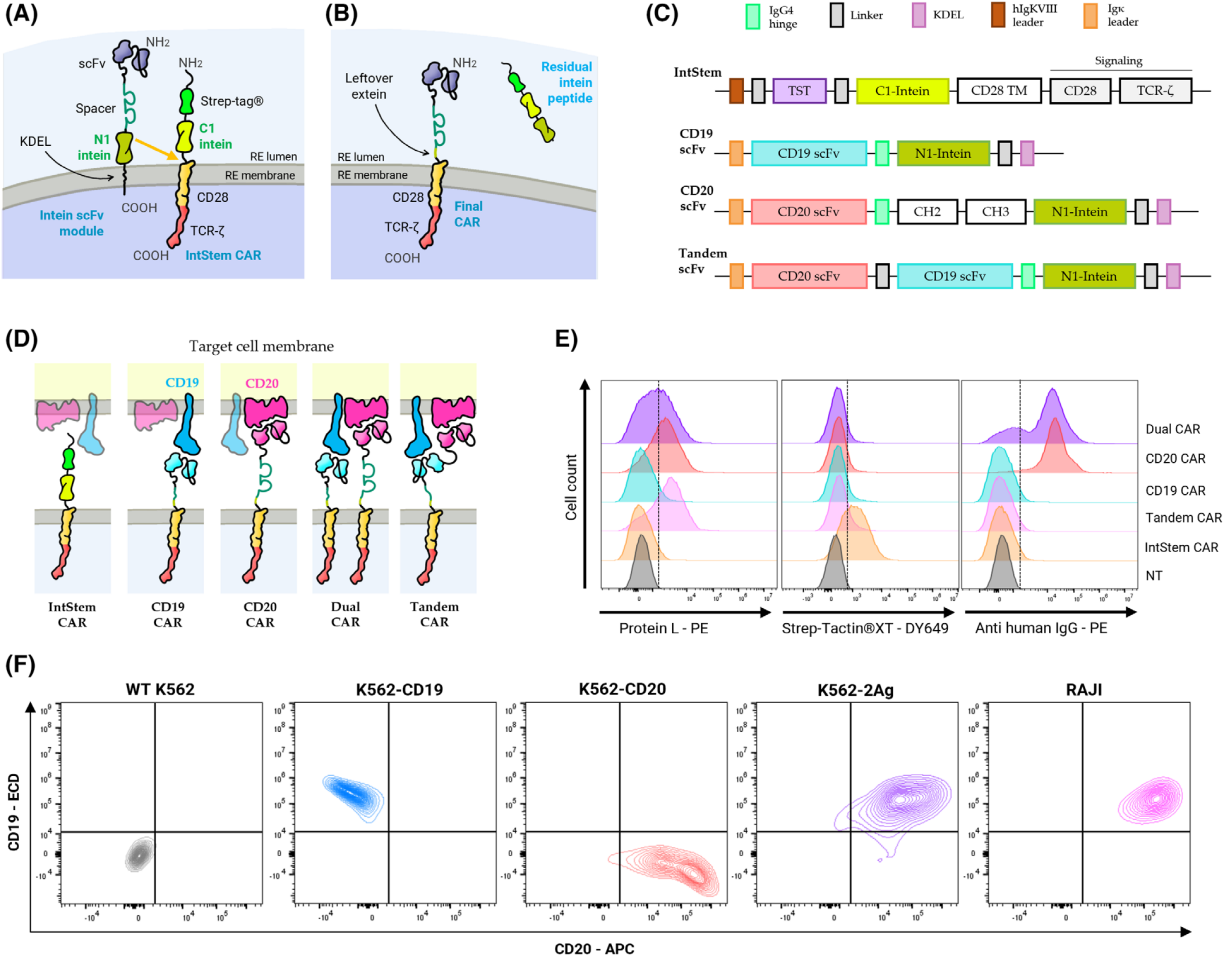
Description of the CARtein platform and surface expression of CD19/CD20 on target cells. (A) Schematic illustration of the CARtein system in the ER, detailing the respective domains of each construct and how they interact. (B) Schematics of the products of the reaction by which a functional CAR is generated, together with an intein by‐product. (C) Schematic representation of each construct used in the manuscript and (D) mature CARtein product on the T cell membrane interacting with their cognate antigens. (E) Evaluation by spectral flow cytometry of CAR expression in Jurkat‐TPR cells and (F) CD19/CD20 expression in the different target cells. Histograms shown are representative of three independent experiments (*n* = 3). NT, non‐transduced; APC, allophycocyanin; ECD, Energy Coupled Dye.

To generate an intein signaling CAR module (IntStem), we used a second‐generation CD28/ζ CAR [17] with a Twin‐Strep‐tag^®^ (TST) for easy identification of unspliced receptors. To enable protein splicing with the different scFv‐intein recognition modules (scFvIRM), we included a C‐intein in the IntStem CAR, while each scFvIRM includes the orthogonal N‐intein. These scFvIRM were expressed in CAR‐TPR cells already expressing IntStem, to allow for the transplicing reaction to occur in the endoplasmic reticulum (ER), where scFvIRM are retained due to a KDEL sequence to prevent scFvIRM secretion of unspliced modules (Fig. 1A). As a result, CARs gain functionality due to the incorporation of the recognition moiety, leaving a small leftover between the scFv and the IntStem CAR transmembrane domain (TMD). As they react above the TMD, it loses its TST peptide, which is carried away by an irrelevant intein peptide by‐product (Fig. 1B).

The design allows us to compare not only anti‐CD19 or anti‐CD20 recognition modules but also anti‐CD19‐CD20 tandem CARs onto the same IntStem. As it has been previously shown that the optimal spacer length in CD19 and CD20 CARs differ due to the differential separation of their cognate antigens from the tumor cell membrane, this approach also allows us to customize recognition modules of different lengths that will assemble onto a common IntStem. Since the CD19 antigen is known to reach farther from the membrane than CD20, which is a multiloop transmembrane protein, it was proposed that CD20‐CARs, require a longer spacer between the TMD and the scFv [18], for which we have chosen IgG_4_ hinge‐CH_2_‐CH_3_ [17]. This Fc domain was mutated to prevent interactions with Fc receptors, thus reducing off‐target activation hazards [22, 23]. On the other hand, a shorter spacer version was used for the anti‐CD19 recognition module, including only the IgG_4_ hinge region. In the case of tandem scFv, the relative position of the antigen was also considered, whereby the CD20‐scFv was placed at the N‐terminus and the CD19‐scFv closer to the hinge‐N‐intein, both joined by a flexible linker (Fig. 1C,D). Efficacy of the reaction was evaluated by loss of the TST peptide, which was expected to be replaced by the different scFvIRM. As seen in Fig. 1E, scFvIRM and IntStem co‐expression, effectively removes TST from IntStem CARs, whereas scFv can be detected by Protein‐L on Tandem, Dual and CD20‐CAR expressing cells. The chosen CD19‐scFv sequence, in contrast, is known to be only mildly detectable by Protein‐L. Moreover, human IgG from the anti‐CD20‐intein construct was also detected on both CD20 and Dual CAR expressing cells but not in cells expressing only CD19 or tandem modules as the hinge region does not contain the epitope. To evaluate the specificity of each recognition module toward its cognate target, K562 cells were transduced with either antigen (CD19 or CD20) or both. Therefore, four different sublines were tested: K562‐*Wild‐Type* (WT) (CD19^−^ CD20^−^), K562‐CD19 (CD19^+^ CD20^−^), K562‐CD20 (CD19^−^ CD20^+^) and K562‐2Ag (CD19^+^ CD20^+^). Expression of these antigens on K562, as well as in Raji cells (CD19^+^ CD20^+^), was assessed by spectral flow cytometry (Fig. 1F).

### 3.2 Evaluation of the specificity of CARteins against individual antigens

**Fig. 2:**
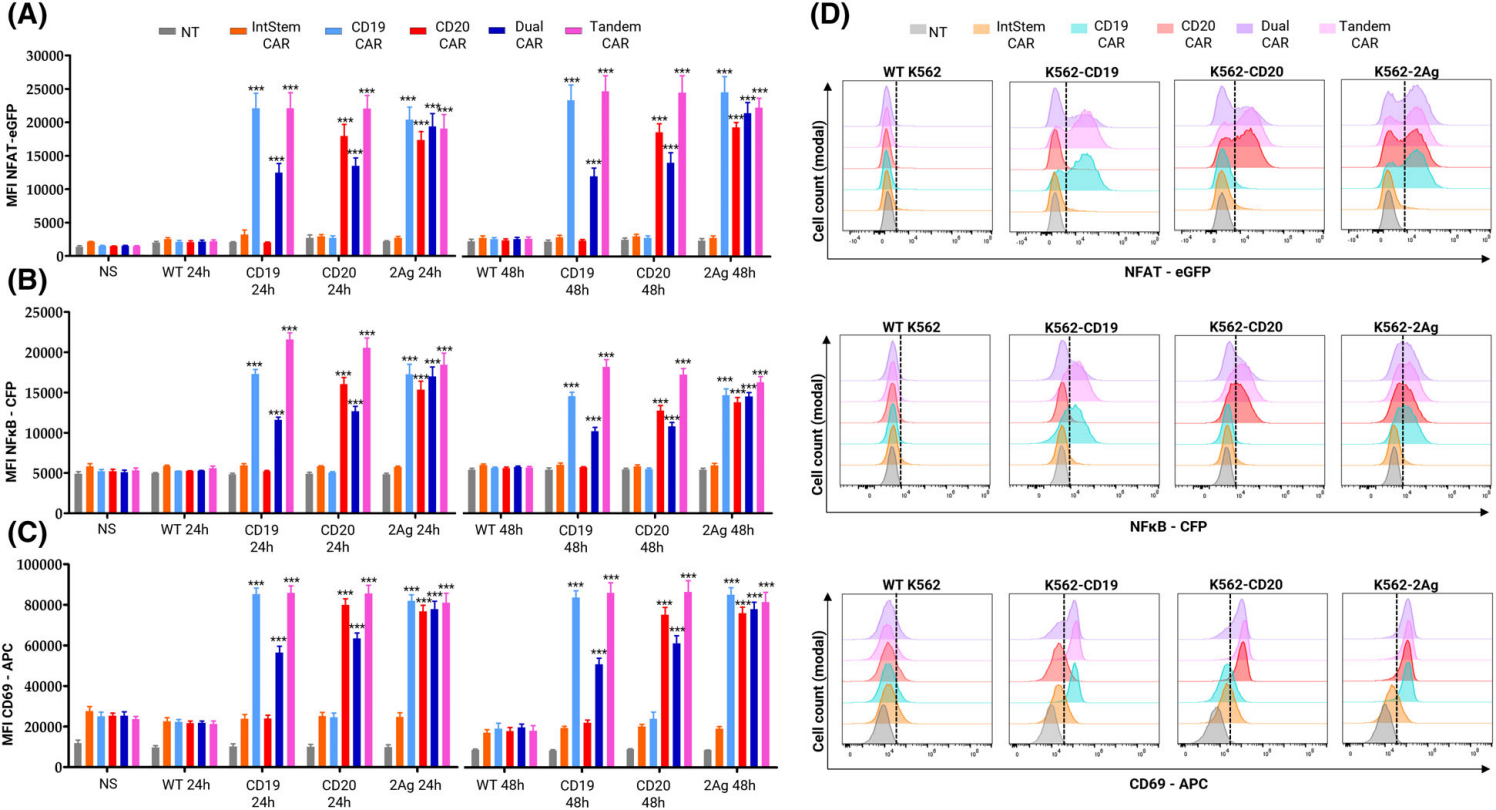
Specific activation of CARtein constructs upon binding of individual antigens. NFAT (eGFP) (A) or NFκB (CFP) (B) promoter activity for each CAR, 24 and 48 h after stimulation with different K562 cell sublines (1:1 ratio), as well as CD69 (APC) upregulation (C) is shown. (D) Representative histograms of each parameter 24 h after stimulation are shown for each condition. Error bars represent mean ± SEM of MFI values from three replicates (*n* = 3). Statistical significance was determined by two‐way ANOVA, with comparisons made against non‐stimulated (NS) cells (***P < 0.001). APC, allophycocyanin; MFI, mean fluorescence intensity; NT, non‐transduced.

To analyze the specificity of this platform, we studied how each CAR‐TPR would respond to K562 cells expressing either CD19, CD20, or both (Fig. 2A–D). NFAT (eGFP; Fig. 2A) and NFκB (CFP; Fig. 2B) reporter activity, together with CD69 upregulation (Fig. 2C), were quantified by spectral flow cytometry, with histograms shown in Fig. 2D. Cells expressing CARs including specific scFv were activated upon binding to its cognate antigen (***P < 0.001 for each parameter, at each time), while remaining unstimulated for the other antigens. Activation of single‐scFv CARteins (CD19‐ and CD20‐CARs), elicited high NFAT and NFκB promoter activity and CD69 upregulation in the presence of K562‐CD19 or K562‐CD20, respectively (***P < 0.001), and both are similarly stimulated with K562‐2Ag (CD19^+^CD20^+^). Activation of Dual CARs is lower to that of Tandem CARs when co‐cultured with single antigen K562 cells, but both are similarly activated by K562‐2Ag. This suggests that the strength of our platform lies not only in scenarios where one of the antigens has been downregulated and needs to be replaced, but also when both target antigens are expressed. In such cases, dual CARs act synergistically when bound to both antigens, resulting in stronger activation, as observed with Raji cells. In contrast, tandem CARs exhibit similar promoter activity and CD69 upregulation regardless of the presence of CD19, CD20, or both. Furthermore, as when co‐cultured with Raji cells, IntStem CAR‐TPR cells do not respond to any K562 cell subline, nor to K562‐WT cells, reinforcing their suitability as inert modules in the absence of the recognition moiety.

### 3.3 CARtein activation upon targeting Raji cells

**Fig. 3:**
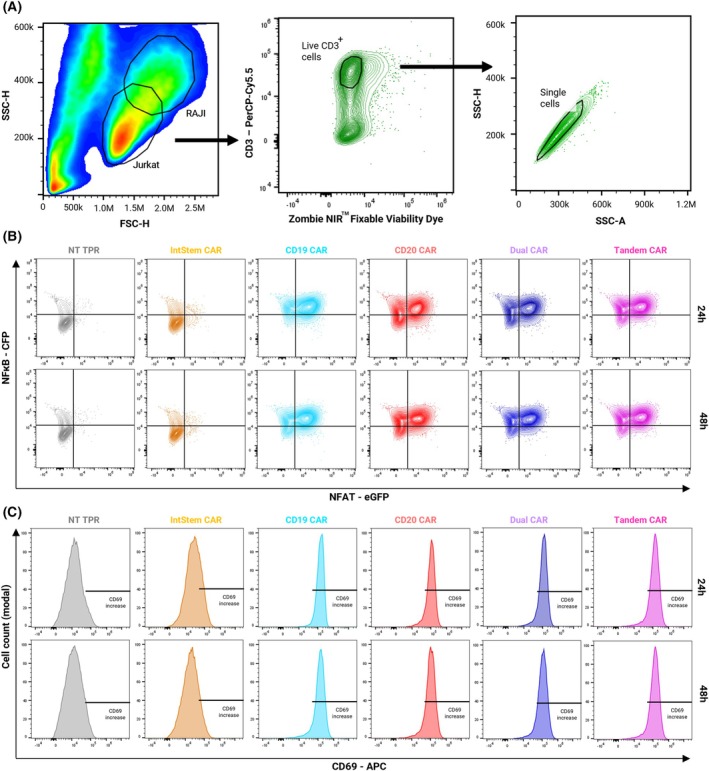
Activation of CAR‐TPR cells upon stimulation with Raji cells. (A) Illustration of the spectral flow cytometry gating strategy, selecting single live CD3+ cells, corresponding to Jurkat‐TPR cells. (B) Dot plots showing transcriptional reporter readouts of NFAT–eGFP and NFκB–CFP fluorescence at 24 h and 48 h after Raji stimulation for the indicated CAR constructs. Quadrants indicate cells activating neither, one, or both promoters. (C) Histograms showing CD69 (APC) expression at 24 h and 48 h after Raji stimulation. Shown histograms are representative of three independent experiments (*n* = 3). APC, allophycocyanin; FSC, Forward Scatter; NT, non‐transduced; SSC, Side Scatter.

Once the response against individual antigens was evaluated, we aimed to test the functionality of the CARtein platform by targeting Raji cells, which have been widely used as a CD19^+^CD20^+^ Burkitt lymphoma cell line model [7]. Cells were co‐cultured at 1:1, 1:5 and 1:10 target:effector ratios and activation was analyzed after 24 and 48 h. Effector cells were distinguished from Raji cells by gating single, live CD3^+^ cells (Fig. 3A). Activation of engineered Jurkat CAR‐TPR cells after co‐culture with Raji targets was quantified by spectral flow cytometry within a standardized single‐live‐cell CD3+ gate to ensure comparable analyses across conditions (Fig. 3A).

Figure 3B displays bivariate dot plots of NFAT–eGFP versus NFκB–CFP at 24 h and 48 h, highlighting cells activating one or both transcriptional reporters upon antigen stimulation. Figure 3C shows histograms of the early activation marker CD69 (APC) at the same time points, demonstrating upregulation relative to non‐transduced controls within the live‐singlet CD3+ gate. Raji cells elicit a basal activation of the NFκB promoter and a subtle upregulation of CD69, but it is negligible when compared to that elicited by CAR‐specific stimulation. Interestingly, co‐culture with Raji cells did not induce NFAT activity in non‐transduced (NT) CAR TPR cells or cells expressing only the IntStem module, while it was highly upregulated in CAR‐expressing cells. As expected, the four different effector CAR cell sublines (CD19, CD20, Dual, and Tandem CARs) are activated when co‐cultured with Raji target cells, manifested by a predominance of the NFAT^+^NFkB^+^ responder population and upregulation of CD69 in >90% of cells.

**Fig. 4:**
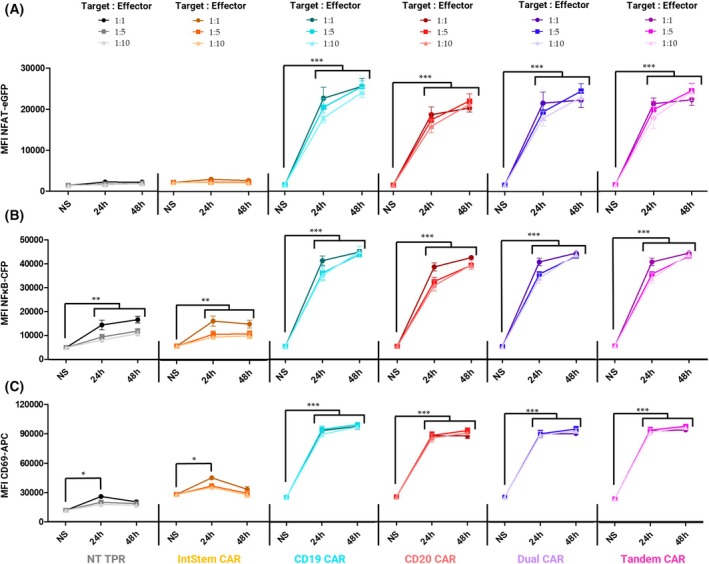
Kinetics of CAR‐TPR activation across time and target‐to‐effector ratios. Mean fluorescence intensity (MFI) for NFAT–eGFP (A), NFκB–CFP (B), or CD69 (APC) (C), measured at non‐stimulated (NS), 24 h, and 48 h after co‐culture with Raji cells at the indicated target:effector ratios. Data are presented as mean ± SEM of MFI from three independent experiments (*n* = 3). Statistical significance was evaluated by two‐way ANOVA with comparisons to NS controls: *P < 0.05, **P < 0.01, ***P < 0.001. APC, allophycocyanin; MFI, mean fluorescence intensity; NT, non‐transduced.

Two‐way ANOVA analysis showed that these increases were statistically significant compared with unstimulated cells (Fig. 4). High NFAT (Fig. 4A) and NFκB (Fig. 4B) promoter activation, as well as CD69 upregulation (Fig. 4C), are evident 24 h after stimulation with Raji cells, although the peak of activation occurs at 48 h, being higher than at 24 h in terms of NFAT and NFκB promoter activation (**P < 0.01), but not for CD69, whose levels in activated cells were already topped 24 h post‐activation. Interestingly, this upward trend is more noticeable at lower target:effector (T:E) ratios, being higher at 1:10 and 1:5 ratios. However, no overall significant differences were observed between the different T:E ratios, even though stimulation strength tends to increase slightly with higher ratios. Of note, IntStem CARs do not respond to Raji stimulation in the absence of any complementary scFvIRM binding partner, thus demonstrating its inert nature. In unstimulated IntStem CAR‐TPR cells, only a weak increase in basal CD69 expression is observed. However, the behavior of these cells is similar to that of non‐transduced TPR cells when co‐cultured with Raji cells, with null NFAT activity but subtle nonspecific NFκB activation and CD69 upregulation so it cannot be explained just by IntStem‐mediated tonic signals.

### 3.4 Protein splicing occurs in primary T cells

**Fig. 5:**
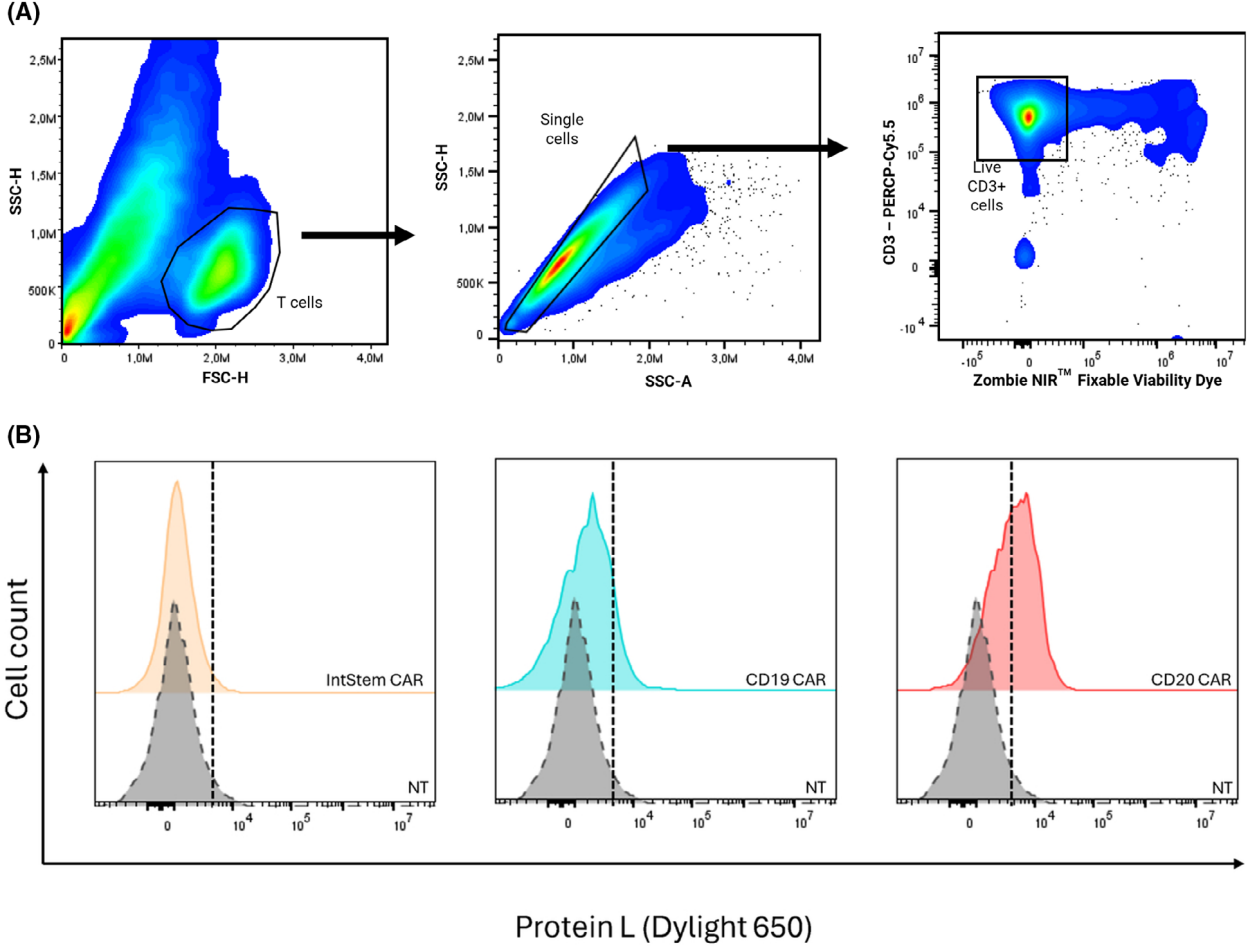
CARtein splicing in primary T cells. (A) Illustration of the spectral flow cytometry gating strategy, selecting single live CD3^+^ cells, which accounts for more than 95% of cells. (B) Evaluation of CARtein expression in T cells, based on protein L staining for cells expressing both signaling and recognition constructs (CD20 CAR or CD19 CAR) compared with cells expressing only the signaling construct (IntStem CAR) and with non‐transduced controls (NT). Histograms shown are representative of three independent stainings (*n* = 3). APC, allophycocyanin; FSC, Forward Scatter; NT, non‐transduced; SSC, Side Scatter.

Having validated the functionality of the modular CAR platform in Jurkat cells, we extended our experiments to primary human T cells to assess the applicability of the system in a clinically relevant context. CD3+ T cells were purified from donor PBMC and subjected to co‐transduction with lentiviral vectors coding for both the signaling module and the antigen‐recognition module. As shown in Fig. 5, cells were first gated on FSC/SSC to identify the T‐cell population, followed by singlet discrimination and exclusion of non‐viable events using a fixable Zombie NIR dye before selecting CD3+ live cells for readouts (Fig. 5A). In Fig. 5B, flow cytometry analysis using protein L staining revealed surface expression exclusively in the population of co‐transduced cells, indicating successful assembly and splicing of the modular CAR. In contrast, primary T cells transduced with only the signaling module exhibited no detectable protein L staining, confirming that antigen‐recognition module delivery is essential for surface expression of the mature receptor. These findings establish that the modular CAR also assembles in primary human T cells, confirming its potential applicability for translational and therapeutic studies.

## 4 Discussion

CAR‐T cell therapy has shown promising results in the fight against lymphomas and leukemias, such as B‐ALL or B‐NHL. In this regard, CD19 is a well‐established target due to its consistent expression in B‐cell neoplasms, although CAR‐CD19 cells often fail in tumor eradication due to loss of the antigen or overgrowth of pre‐existing CD19^−^ malignant cell subsets, for which additional antigens, such as CD20, have been proposed as potential targets. Therefore, multiple targeting has emerged as an interesting alternative to conventional therapies, from which the concept of modular CARs rapidly emerged and gained appeal in the clinic. Modular CARs allow not only to bind multiple individual scFv, but also several tandem CARs, thus broadening the spectrum of potential targets. In this study, we have demonstrated that the design of a modular CAR platform based on intein interaction is feasible and efficient, allowing T cells to express an intein‐based modular backbone (IntStem CAR) to which different scFvIRM can be attached. We have chosen IMPDH‐1 intein as it exhibits the highest trans‐splicing rates and yields [24, 25], and it has already been shown to function in ER transplicing [25]. This IntStem CAR alone was also shown to be inert, with low tonic signaling that was only perceived by a subtle CD69 upregulation. However, this was similar to what was observed with CD19, CD20, and Dual or Tandem CARs, all of them sharing the CD28 transmembrane domain (CD28‐TMD), that has already been associated with tonic signaling [26].

Our results demonstrate that Jurkat T cells expressing mature transpliced CARs are highly sensitive to antigen‐specific stimulation when expressing the CD19/CD20 CARtein platform. This is evidenced by the marked upregulation of CD69 and significant NFAT/NFκB promoter activities only in IntStem CAR‐TPR cells co‐transduced with their respective scFvIRM‐binding proteins and co‐cultured with their cognate target cells. Platform specificity was further confirmed by exposing these effector cells to K562 cells expressing CD19 and/or CD20. When targeting Raji cells, CAR‐TPR activation is sufficiently strong regardless of the effector‐to‐target ratio, although they show a tendency to be slightly higher at higher ratios. Moreover, activation is also time‐dependent, peaking at 48 h after stimulation. This agrees with our previous work showing that a CAR‐like construct with the same CD28/ζ backbone has its global peak activity 48 h after stimulation, with peaks obtained at later time points for weaker CAR‐antigen interactions [17]. This finding would explain why both NFκB and NFAT activities stabilize 48 h after stimulation at higher E:T ratios but has yet to reach it peak at this time point for lower ratios.

Loss of STT peptide and gain of IgG spacer and/or scFv reveals the efficacy of scFvIRM binding to the IntStem CAR backbone. In the case of Dual CARs, in which CD19‐ and CD20‐scFvIRM have been co‐transduced, the readiness of the IntStem to bind more than one receptor in the same cell is also shown. However, these cells show a lower activation when only one antigen is present in target cells, which could be explained by the fact that in Dual CARs, the IntStem backbone is equally occupied by both scFvIRM, so that in practice, they express half as many CD19‐CARs and half as many CD20‐CARs as their single‐scFvIRM counterparts, with half the constructs being idle against a single antigen target. Furthermore, Dual CARs promote activation similar to that of Tandem CARs when co‐cultured with CD19^+^CD20^+^ cells, such as Raji and K562‐2Ag, since in both conditions CARs are activated equally by CD19 or CD20. Thus, Tandem CAR acts as an AND/OR Boolean logic gate to exert high stimulation, by which it only needs to recognize one antigen, whereby this Dual modular CAR would require an CD19‐AND‐CD20 logic gate to reach its maximal activity.

Interestingly, NFAT promoter activity is null in both non‐transduced CAR‐TPR (NT) and IntStem cells in the presence of Raji cells but was significantly elevated in stimulated CAR‐TPR cells. Thus, in this scenario, the NFAT promoter is more specific to the TCR/CD28 axis than other parameters such as CD69 upregulation and NFκB activity, which are slightly increased in both NT and IntStem CAR‐TPR cells co‐cultured with Raji cells. This could be explained by the expression of costimulatory molecules on the surface of Raji cells, such as CD70 and CD80/CD86, which are known to promote NFκB activity in T cells [27, 28]. This, in addition to the fact that K562 cells do not express these molecules [29], could be the reason for the null NFκB activity of TPR cells when co‐cultured with K562 cells.

Finally, while the foundational molecular validation of our intein‐mediated system in Jurkat cells provided essential mechanistic insights and proof‐of‐concept data, we demonstrated splicing in primary human T cells. Accordingly, upon obtaining IRB approval, we performed co‐transduction experiments in primary CD3+ T cells, confirming successful modular CAR assembly. These results reinforce the translational relevance of the CARtein approach and establish a framework for broader clinical translation. In clinical settings, co‐transduction of primary T cells with modular constructs followed by antigen‐driven expansion, will ensure efficient integration of the desired components and selective enrichment of mature CAR‐T expressing populations. A noteworthy advantage of the our modular paradigm is the flexibility afforded by administering the antigen recognition module in trans, after initial transduction of the signaling module. This strategy, underlying so‐called universal or “switchable” CAR platforms, enables clinicians to adjust dosing or halt the administration of antigen recognition modules in response to patient needs or adverse events, offering a degree of control not achievable with conventional CAR constructs. From a regulatory perspective, the most effective pathway initially will involve submitting the complete modular system, including both signaling and antigen recognition modules as a unified medicinal product. This coordinated approval allows for parallel risk assessment and establishes a robust safety and efficacy profile for the integrated CAR‐T technology as intended for clinical use. Subsequent applications targeting alternative antigens, but utilizing the validated signaling backbone, can often leverage this established regulatory foundation, facilitating streamlined approvals and mitigating the need to readdress previously resolved safety questions. This modular registration philosophy is increasingly reflected in the evolving regulatory landscape for customizable cell therapies, supporting both innovation and patient safety while promoting scalability.

## 5 Conclusions

In conclusion, our study lays the groundwork for the construction of modular CARs based on intein interactions. This innovative approach, which is an interesting alternative to conventional binding partners, involves a universal CAR backbone that covalently binds to its cognate scFvIRM, resulting in a fully active receptor with a very similar structure to that of a conventional CAR. The CARtein CD19/CD20 system exemplifies the potential of this modular platform to target multiple molecules, thereby addressing challenges such as antigen escape and tumor heterogeneity in B‐cell neoplasms. Furthermore, the inert nature of IntStem‐CARs underscores the accuracy and safety of this approach. Moreover, our work constitutes a proof of concept for a new modular CAR platform that could be tailored by adding different recognition modules depending on the antigen repertoire of each patient's tumor cell.

## Conflict of interest

All authors are inventors of the patent application EP25382080.7, filed by the University of Cádiz and INIBICA. The patent pertains to modular CARs, and uses thereof, which is directly related to the findings reported in this manuscript. No financial or non‐financial benefits have been realized from this patent at the time of publication. The authors confirm that no other competing interests exist.

## Author contributions

FGC contributed to conceptualization, funding acquisition, supervision, validation, project administration, writing review, and editing. PGG, NM and WYH were responsible for conceptualization, investigation, data curation, visualization, and writing original draft preparation. WYH and RFC performed the formal analysis. JOC contributed to methodology development. JPMM, AG, and RLE performed data acquisition and developed reagents essential for the study. CMFP assisted with validation. All authors reviewed and approved the final manuscript.

## Data Availability

The data that support the findings of this study are available from the corresponding author [curro.garcia@uca.es] upon reasonable request.

## References

[1] Makita S , Tobinai K . Antibody therapy targeting CD19 for B‐cell non‐Hodgkin's lymphoma. Ann Oncol. 2018;29(5):1086–1089. 29554220 10.1093/annonc/mdy092

[2] Davila ML , Brentjens RJ . CD19‐targeted CAR T cells as novel cancer immunotherapy for relapsed or refractory B‐cell acute lymphoblastic leukemia. Clin Adv Hematol Oncol. 2016;14(10):802–808. 27930631 PMC5536094

[3] Ostojska M , Nowak E , Twardowska J , Lejman M , Zawitkowska J . CAR‐T cell therapy in the treatment of pediatric non‐Hodgkin lymphoma. J Pers Med. 2023;13(11):1595. 38003910 10.3390/jpm13111595 PMC10672004

[4] Todorovic Z , Todorovic D , Markovic V , Ladjevac N , Zdravkovic N , Djurdjevic P , et al. CAR T cell therapy for chronic lymphocytic leukemia: successes and shortcomings. Curr Oncol. 2022;29(5):3647–3657. 35621683 10.3390/curroncol29050293 PMC9139644

[5] Xu X , Sun Q , Liang X , Chen Z , Zhang X , Zhou X , et al. Mechanisms of relapse after CD19 CAR T‐cell therapy for acute lymphoblastic leukemia and its prevention and treatment strategies. Front Immunol. 2019;10:2664. 31798590 10.3389/fimmu.2019.02664 PMC6863137

[6] Cordoba S , Onuoha S , Thomas S , Pignataro DS , Hough R , Ghorashian S , et al. CAR T cells with dual targeting of CD19 and CD22 in pediatric and young adult patients with relapsed or refractory B cell acute lymphoblastic leukemia: a phase 1 trial. Nat Med. 2021;27(10):1797–1805. 34642489 10.1038/s41591-021-01497-1 PMC8516648

[7] Yang N , Zhang C , Zhang Y , Fan Y , Zhang J , Lin X , et al. CD19/CD20 dual‐targeted chimeric antigen receptor‐engineered natural killer cells exhibit improved cytotoxicity against acute lymphoblastic leukemia. J Transl Med. 2024;22(1):274. 38475814 10.1186/s12967-024-04990-6 PMC10935961

[8] Tong C , Zhang Y , Liu Y , Ji X , Zhang W , Guo Y , et al. Optimized tandem CD19/CD20 CAR‐engineered T cells in refractory/relapsed B‐cell lymphoma. Blood. 2020;136(14):1632–1644. 32556247 10.1182/blood.2020005278 PMC7596761

[9] Fang C , Zhuang Y , Wang L , Fan L , Wu Y , Zhang R , et al. High levels of CD20 expression predict good prognosis in chronic lymphocytic leukemia. Cancer Sci. 2013;104(8):996–1001. 23659384 10.1111/cas.12192 PMC7657116

[10] Kaleem Z , Hassan A , Pathan MH , White G . Flow cytometric evaluation of posttransplant B‐cell lymphoproliferative disorders. Arch Pathol Lab Med. 2004;128(2):181–186. 14736286 10.5858/2004-128-181-FCEOPB

[11] Schneider D , Xiong Y , Wu D , Nölle V , Schmitz S , Haso W , et al. A tandem CD19/CD20 CAR lentiviral vector drives on‐target and off‐target antigen modulation in leukemia cell lines. J Immunother Cancer. 2017;5(1):42. 28515942 10.1186/s40425-017-0246-1 PMC5433150

[12] McCue AC , Yao Z , Kuhlman B . Advances in modular control of CAR‐T therapy with adapter‐mediated CARs. Adv Drug Deliv Rev. 2022;187:114358. 35618140 10.1016/j.addr.2022.114358 PMC9939278

[13] Sutherland AR , Owens MN , Geyer CR . Modular chimeric antigen receptor systems for universal CAR T cell retargeting. Int J Mol Sci. 2020;21(19):7222. 33007850 10.3390/ijms21197222 PMC7582510

[14] Shah NH , Muir TW . Inteins: nature's gift to protein chemists. Chem Sci. 2013;5(2):446–461. 10.1039/C3SC52951G PMC3949740 24634716

[15] Wang H , Wang L , Zhong B , Dai Z . Protein splicing of Inteins: a powerful tool in synthetic biology. Front Bioeng Biotechnol. 2022;10:810180. 35265596 10.3389/fbioe.2022.810180 PMC8899391

[16] Ramsden R , Arms L , Davis TN , Muller EG . An intein with genetically selectable markers provides a new approach to internally label proteins with GFP. BMC Biotechnol. 2011;11(1):71. 21708017 10.1186/1472-6750-11-71 PMC3141402

[17] Gonzalez‐Garcia P , Muñoz‐Miranda JP , Fernandez‐Cisnal R , Olvera L , Moares N , Gabucio A , et al. Specific activation of T cells by an ACE2‐based CAR‐like receptor upon recognition of SARS‐CoV‐2 spike protein. Int J Mol Sci. 2023;24:7641. 37108807 10.3390/ijms24087641 PMC10145580

[18] Zah E , Lin M‐Y , Silva‐Benedict A , Jensen MC , Chen YY . T cells expressing CD19/CD20 bispecific chimeric antigen receptors prevent antigen escape by malignant B cells. Cancer Immunol Res. 2016;4(6):498–508. 27059623 10.1158/2326-6066.CIR-15-0231 PMC4933590

[19] Hamilton JR , Chen E , Perez BS , Espinoza CRS , Kang MH , Trinidad M , et al. In vivo human T cell engineering with enveloped delivery vehicles. Nat Biotechnol. 2024;1:1–9. 10.1038/s41587-023-02085-z PMC11236958 38212493

[20] Rodriguez‐Marquez P , Calleja‐Cervantes ME , Serrano G , Oliver‐Caldes A , Palacios‐Berraquero ML , Martin‐Mallo A , et al. CAR density influences antitumoral efficacy of BCMA CAR T cells and correlates with clinical outcome. *medRxiv* . 10.1126/sciadv.abo0514 PMC9524842 36179026

[21] Tang Y , Garson K , Li L , Vanderhyden BC . Optimization of lentiviral vector production using polyethylenimine‐mediated transfection. Oncol Lett. 2015;9(1):55–62. 25435933 10.3892/ol.2014.2684 PMC4246624

[22] Hombach A , Hombach AA , Abken H . Adoptive immunotherapy with genetically engineered T cells: modification of the IgG1 fc ‘spacer’ domain in the extracellular moiety of chimeric antigen receptors avoids ‘off‐target’ activation and unintended initiation of an innate immune response. Gene Ther. 2010;17(10):1206–1213. 20555360 10.1038/gt.2010.91

[23] Wang H , Tang L , Kong Y , Liu W , Zhu X , You Y . Strategies for reducing toxicity and enhancing efficacy of chimeric antigen receptor T cell therapy in hematological malignancies. Int J Mol Sci. 2023;24(11):9115. 37298069 10.3390/ijms24119115 PMC10252534

[24] Carvajal‐Vallejos P , Pallissé R , Mootz HD , Schmidt SR . Unprecedented rates and efficiencies revealed for new natural Split Inteins from metagenomic sources. J Biol Chem. 2012;287(34):28686–28696. 22753413 10.1074/jbc.M112.372680 PMC3436554

[25] Moll JM , Wehmöller M , Frank NC , Homey L , Baran P , Garbers C , et al. Split2 protein‐ligation generates active IL‐6‐type hyper‐cytokines from inactive precursors. ACS Synth Biol. 2017;6(12):2260–2272. 29136368 10.1021/acssynbio.7b00208

[26] Ferreira LMR , Muller YD . CAR T‐cell therapy: is CD28‐CAR heterodimerization its Achilles' heel? Front Immunol. 2021;12:766220. 34868017 10.3389/fimmu.2021.766220 PMC8635711

[27] Han BK , Olsen NJ , Bottaro A . The CD27–CD70 pathway and pathogenesis of autoimmune disease. Semin Arthritis Rheum. 2016;45(4):496–501. 26359318 10.1016/j.semarthrit.2015.08.001

[28] Thaker YR , Schneider H , Rudd CE . TCR and CD28 activate the transcription factor NF‐κB in T‐cells via distinct adaptor signaling complexes. Immunol Lett. 2015;163(1):113–119. 25455592 10.1016/j.imlet.2014.10.020 PMC4286576

[29] Butler MO , Hirano N . Human cell‐based artificial antigen‐presenting cells for cancer immunotherapy. Immunol Rev. 2014;257(1):191–209. 24329798 10.1111/imr.12129 PMC3869003

